# Pseudoexfoliation Syndrome Presenting With Bilateral Visual Impairment and Intraocular Pressure Discrepancy: A Case Report

**DOI:** 10.7759/cureus.56450

**Published:** 2024-03-19

**Authors:** Devwrath Upasani, Sachin Daigavane

**Affiliations:** 1 Ophthalmology, Jawaharlal Nehru Medical College, Datta Meghe Institute of Higher Education & Research, Wardha, IND

**Keywords:** surgical intervention, glaucoma, cataract extraction, intraocular pressure discrepancy, bilateral visual impairment, pseudoexfoliation syndrome

## Abstract

Pseudoexfoliation syndrome (PEX) presents a significant clinical challenge due to its diverse ocular manifestations, including glaucoma and zonular weakness of the lens, which can lead to irreversible visual impairment if left untreated. We report a case of a 78-year-old male presenting with bilateral visual impairment persisting for four years, with a more pronounced decline in the right eye over the past six months. Examination revealed aphakia with pseudoexfoliative material in the right eye, a cataract with pseudoexfoliative material in the left eye, and a notable intraocular pressure (IOP) discrepancy. Surgical intervention was required for both eyes, with cataract extraction and IOP-lowering procedures performed to preserve vision and prevent further deterioration. This case underscores the importance of timely recognition and comprehensive management of PEX-related ocular complications to optimize visual outcomes and quality of life for affected individuals. Close collaboration between ophthalmologists and other healthcare professionals is essential to address the multifaceted nature of PEX and tailoring treatment strategies to individual patient needs. Further research is needed to elucidate the underlying pathophysiology of PEX and refine therapeutic approaches to mitigate its detrimental effects on vision.

## Introduction

Pseudoexfoliation syndrome (PEX) is a systemic disorder characterized by the abnormal deposition of fibrillar material in various ocular tissues, particularly the anterior segment of the eye. Lindberg first described the condition in 1917 as “fibrillopathia epitheliocapsularis” [1]. PEX is associated with advanced age and is more prevalent in individuals over 60 [2]. The etiology of PEX remains unclear, but genetic predisposition and environmental factors such as ultraviolet light exposure and oxidative stress are believed to contribute to its pathogenesis [3]. PEX material comprises fibrillar aggregates of glycoproteins, including elastin and microfibrillar-associated proteins [4].

Clinical manifestations of PEX include the deposition of characteristic white, dandruff-like material on the anterior lens capsule, iris, trabecular meshwork, and other ocular structures [5]. PEX material can lead to complications, including zonular weakness, lens subluxation, and glaucoma [6]. Glaucoma is one of the most significant complications associated with PEX, with approximately 25-50% of patients developing elevated intraocular pressure (IOP) and subsequent optic nerve damage [7]. The mechanism of PEX-related glaucoma involves obstruction of the trabecular meshwork by exfoliative material, leading to impaired aqueous humor outflow and increased IOP [8].

Management of PEX-related glaucoma typically involves medical therapy with topical antiglaucoma medications to lower IOP. However, surgical interventions such as trabeculectomy, glaucoma drainage devices, and laser trabeculoplasty may be required in uncontrolled or advanced disease [9]. Cataract surgery in patients with PEX poses additional challenges due to zonular weakness and the risk of intraoperative complications such as capsular rupture and vitreous loss [10]. Various intraocular lens (IOL) implantation techniques, including iris claw and scleral-fixated IOL, may address these challenges and achieve optimal visual outcomes [11]. Early recognition and management of PEX-related ocular complications are essential to prevent irreversible visual loss and preserve visual function in affected individuals. Multidisciplinary collaboration between ophthalmologists, optometrists, and other healthcare professionals is necessary for the comprehensive care of patients with PEX.

## Case presentation

**Figure 1:**
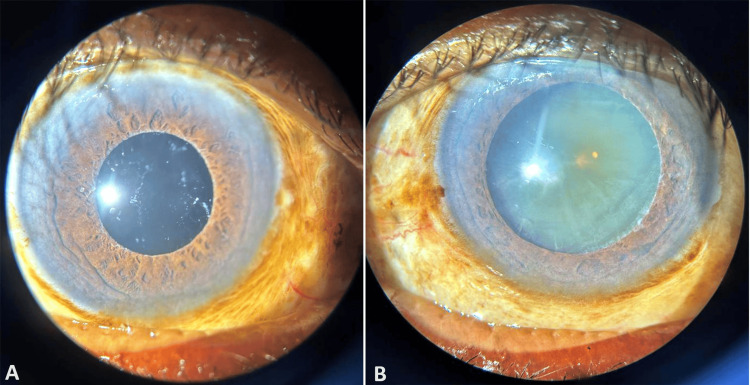
(A) Pseudoexfoliative material at the pupillary margin with aphakia. (B) Pseudoexfoliative material at the pupillary margin with cataract

A 78-year-old male presented to the ophthalmology outpatient department with chief complaints of reduced visual acuity in both eyes for four years. He noted a more pronounced decline in visual acuity in his right eye compared to his left, particularly worsening over the past six months. The patient denied any history of ocular pain or trauma. Upon examination, the patient’s visual acuity was limited to light perception with a projection of rays in the right eye and counting fingers at three meters in the left eye. Slit lamp evaluation revealed aphakia with pseudoexfoliative material at the pupillary margin in the right eye (Figure 1A) and a cataract with pseudoexfoliative material at the pupillary margin in the left eye (Figure 1B). IOP measurements were 15 mmHg in the right eye and elevated at 47 mmHg in the left.

**Figure 2:**
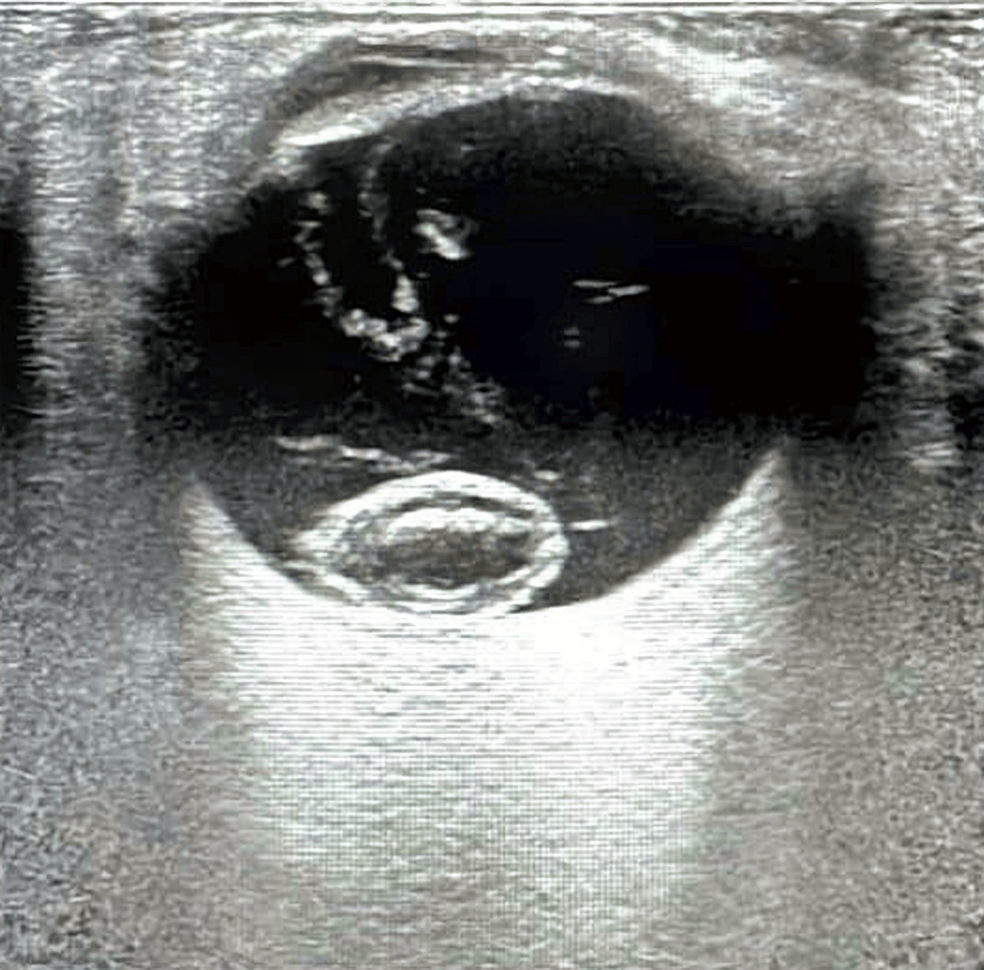
The B-scan shows a lens drop in the vitreous cavity of the right eye

The B-scan ultrasonography confirmed a lens drop in the vitreous cavity of the right eye (Figure 2), while the left eye appeared within normal limits. Fundus photography revealed a lens drop in the right eye and glaucomatous optic disc cupping with a cup-to-disc ratio of 0.8 in the left eye. Systemic examinations were unremarkable, including cardiovascular, pulmonary, and central nervous system evaluations.

**Figure 3:**
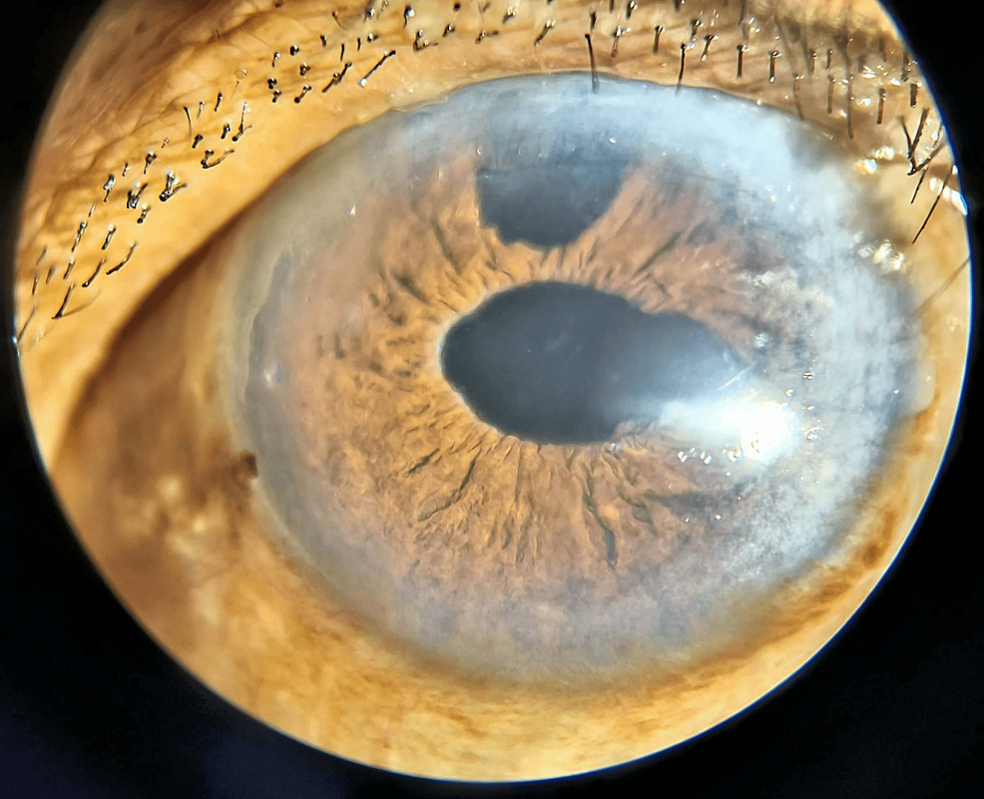
Iris claw implantation in the left eye

**Figure 4:**
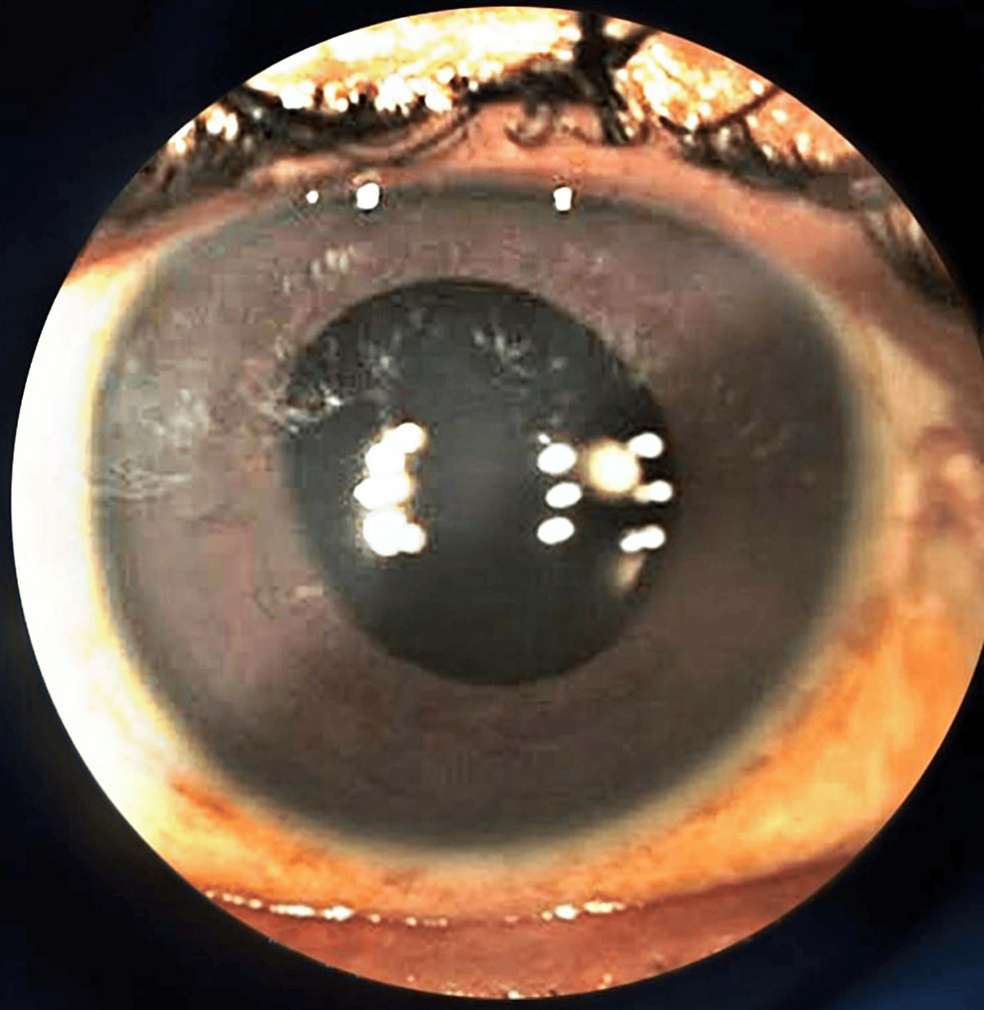
Scleral fixation of IOL implantation in the right eye IOL, intraocular lens

Treatment commenced with acetazolamide 250 mg tablets twice daily and eye drops containing timolol (0.5%) and brimonidine (0.15%) to reduce IOP in the left eye. Written informed consent was obtained for cataract extraction in the left eye, during which zonular weakness and phacodonesis were noted. Due to posterior capsule integrity issues, posterior chamber IOL implantation was not feasible, necessitating iris claw implantation in the left eye (Figure 3). Follow-up after two weeks focused on the right eye, leading to informed consent for pars plana vitrectomy with lens explantation followed by scleral-fixated IOL implantation in the right eye (Figure 4). Both eyes exhibited PEX.

The patient was followed up after one month postoperatively. The anterior chamber was well formed and quiet in both eyes. The visual acuity in the right eye improved to 6/36, and that of the left eye improved to 6/24. The patient was discharged postoperatively with eye drop timolol (0.5%) plus brimonidine (0.15%). On follow-up, the IOP was well controlled: the IOP in the right eye was 18 mmHg, and that of the left eye was 16 mmHg during the one-month follow-up. The patient was advised to follow up on the OPD monthly to check the IOP changes. The patient had a favorable prognosis despite undergoing secondary IOL implantation in both eyes, with a scleral-fixated IOL in the right eye and an iris claw in the left eye. The main challenge in such cases is control of IOP; in this case, it was well under control, so there was no need to add an extra antiglaucoma drug.

## Discussion

PEX is a systemic disorder characterized by the abnormal accumulation of fibrillar material throughout the anterior segment of the eye, particularly on the lens capsule and pupillary margin [12]. It is associated with various ocular complications, including cataract formation, glaucoma, zonular weakness, and lens subluxation [13]. The prevalence of PEX varies geographically, with higher rates reported in specific populations, such as those of Scandinavian descent [14]. In the presented case, the patient exhibited bilateral visual impairment secondary to cataract formation and elevated IOP, with a more pronounced presentation in the right eye. This is consistent with previous reports indicating that PEX can lead to asymmetric involvement of the eyes, with one eye often being more severely affected than the other [15]. The mechanism underlying this asymmetry remains unclear but may involve differences in the degree of trabecular meshwork obstruction, variations in the extent of zonular weakness, or variations in the response to oxidative stress [16].

Elevated IOP is a common finding in PEX and is primarily attributed to the development of pseudoexfoliative glaucoma (PXG) [17]. PXG is characterized by progressive optic nerve damage and visual field loss, often leading to irreversible blindness if left untreated [18]. In the present case, the patient exhibited a significant discrepancy in IOP between the two eyes, highlighting the importance of thorough evaluation and management of glaucoma in PEX patients. Treatment strategies for PEX-related glaucoma typically involve topical antiglaucoma medications, such as beta-blockers, prostaglandin analogues, and carbonic anhydrase inhibitors, to lower IOP [19]. In cases of advanced glaucoma or an inadequate response to medical therapy, surgical intervention may be necessary. Surgical options include trabeculectomy, glaucoma drainage devices, and minimally invasive surgeries [20]. Cataract extraction is another common intervention in PEX patients, particularly in cases where visual impairment is primarily attributable to cataract formation [21]. However, surgical management of cataracts in PEX patients can be challenging due to zonular weakness and capsular instability [22]. In the present case, iris claw implantation was performed in the left eye due to posterior capsule integrity issues, highlighting the need for careful surgical planning and intraoperative decision-making in these patients.

## Conclusions

This case report highlights the intricate challenges associated with PEX, particularly its manifestation with bilateral visual impairment and IOP discrepancy. PEX is a multifaceted disorder with ocular complications ranging from zonular weakness of the lens to glaucoma, often leading to significant visual morbidity if left untreated. The presented case emphasizes the importance of vigilant clinical assessment and timely intervention to address the cataractous changes and glaucomatous pathology associated with PEX. Surgical management, including cataract extraction and IOP-lowering procedures, is pivotal in preserving vision and preventing irreversible blindness in affected individuals. Collaboration among ophthalmologists and other healthcare professionals is imperative to deliver comprehensive care tailored to the individual needs of PEX patients. Further research is warranted to elucidate the underlying mechanisms of PEX and refine treatment strategies to optimize visual outcomes and quality of life for affected individuals.
